# Not who we treat, but how we treat: Emphasizing treatment adherence in neuromodulation outcomes

**DOI:** 10.1016/j.inpm.2026.100773

**Published:** 2026-05-22

**Authors:** Ryan S. D'Souza, David Hao, Salim M. Hayek

**Affiliations:** a Department of Anesthesiology and Perioperative Medicine, Mayo Clinic, Rochester, MN, USA; b Department of Anesthesiology, Critical Care, and Pain Medicine, Massachusetts General Hospital, Boston, MA, USA; c Department of Anesthesiology and Perioperative Medicine, Case Western University School of Medicine, University Hospitals Cleveland Medical Center, Cleveland, OH, USA

## 1 Introduction

A simple but often underappreciated principle underlies much of medicine: treatments usually work when they are taken correctly. Antihypertensives lower blood pressure when hypertensive patients take them consistently. Insulin achieves euglycemia in diabetics when administered in the right doses, day after day. Antibiotics eradicate infection when the full prescribed course is completed. Across these seemingly disparate conditions, therapeutic success depends not merely on selecting the right patient, but on delivering the right treatment with appropriate consistency and adherence. This principle, fundamental across chronic disease management [1], is equally paramount in neuromodulation for chronic pain, an area that has traditionally emphasized patient selection but may increasingly warrant a shift toward treatment delivery and adherence.

In this context, the *post-hoc* per-protocol analysis of the RESET trial [2] provides timely insight into outcomes associated with percutaneous 60-day peripheral nerve stimulation (PNS) for chronic low back pain. Building on the randomized controlled trial [3], which suggested superiority of PNS over usual care with standard interventional management, the *post-hoc* analysis [2] explored whether per-protocol treatment delivery and baseline clinical variables were associated with response. Of the 93 participants who completed the 60-day PNS treatment period, 67 (72%) were treatment-adherent by meeting per-protocol criteria while 26 (28%) did not. The key finding was that per-protocol treatment delivery, defined as patient-reported adequate stimulation coverage of the painful area and 6–12 hours of daily stimulator use, was associated with 3.04 greater odds of achieving ≥50% pain reduction at 3 months. Further, baseline variables including age, pain duration, sex, pain intensity, disability, and response to medial branch block, were not associated with outcomes. Taken together, these findings raise two intertwined questions: whether per-protocol delivery causally shapes response to neurostimulation, and whether the observed association reflects the same patient-level factors that drive adherence to any therapy.

## 2 Interpretation and major implications

The primary take-home point from this study is that adherence to appropriate per-protocol delivery of PNS therapy plays a critical role in achieving analgesia. Unlike prior PNS trials that aggregate heterogeneous pain conditions [4,5], this analysis focused on chronic axial low back pain (persistent spinal pain syndrome type 1). Additional strengths include the use of a multicenter dataset, enhancing external validity; incorporation of multivariable analyses to address potential confounding; and a clinically meaningful, reproducible definition of treatment delivery, improving real-world application.

However, this conclusion warrants careful interpretation. Per-protocol analyses inherently capture a subset of patients who successfully adhered to treatment, thereby introducing selection bias. Further, the exposure variable of adherence cannot be randomized. Patients who adhere to therapy may be more likely to be motivated, engaged, possess more favorable expectations, and may differ systematically from those who do not complete treatment as prescribed. As such, the observed association reflects outcomes under ideal rather than real-world conditions, and reinforces the importance of how treatment is delivered and strictly followed.

Indeed, patient disposition which encompasses treatment adherence and completion is recognized by the Initiative on Methods, Measurement, and Pain Assessment in Clinical Trials (IMMPACT) as a core domain in chronic pain research [6,7]. This framework conceptualizes adherence as an outcome, rather than as an exposure variable, that reflects treatment acceptability, tolerability, and patient engagement. Across chronic medical conditions, adherence is strongly associated with improved outcomes [[8], [9], [10]]but this relationship is bidirectional: patients who improve are more likely to remain adherent, while those with persistent symptoms may discontinue therapy. This interdependence creates collinearity between adherence and outcomes such as pain, making it difficult to determine whether adherence drives pain relief or pain relief reinforces adherence. As such, the observed association between per-protocol treatment delivery and analgesia may, in part, reflect reverse causation or shared underlying factors rather than a purely causal effect of treatment adherence.

## 3 The absence of predictors is not evidence of broad applicability

The absence of baseline predictors should not be interpreted as evidence that PNS is broadly applicable and effective across all patients with chronic low back pain. More likely, it reflects the limits of the study design. The analysis was underpowered to detect subtle effects, and the range of clinical variables was constrained by inclusion criteria that already selected for age (≥21 and ≤ 75 years), pain duration (longer than 6 months), and baseline pain severity (≥4 on a scale of 0-10), effectively pre-filtering several of the very predictors under investigation. Moreover, several tested variables are not typically central to real-world patient selection for PNS. Key domains known to influence chronic pain outcomes, such as pain catastrophizing, self-efficacy, mental health, and prior opioid use, were not assessed. Future studies should move toward multidimensional models that better reflect the biopsychosocial nature of chronic pain, rather than relying on a limited set of baseline characteristics.

## 4 Limitations and biases

Additionally, a number of limitations were prevalent in the parent (RESET) study [3] including the absence of a sham control arm, expectation bias given unblinded treatment allocation and involvement of industry personnel in patient programming, and absence of standardization in the “standard interventional management” arm of the study. To the extent that these factors act jointly on the per-protocol delivery and outcome variables, the magnitude of the adherence-response association is plausibly overestimated in this industry-sponsored intervention.

## 5 Reconsideration of the prognostic role of nerve blocks

The lack of association between medial branch block response and PNS outcomes should be interpreted with caution. While this finding adds to growing skepticism around the prognostic value of diagnostic nerve blocks in neuromodulation, it is far from definitive. The analysis relied on a single-block paradigm, incorporated both pre-enrollment and protocolized blocks, and included incomplete diary data. Moreover, existing literature suggests that diagnostic blocks may have context-dependent value, particularly for short-term response after temporary PNS [11], although evidence remains mixed [12]. Importantly, the role of diagnostic blocks may extend beyond its questionable predictive value. In practice, they can help refine pain generators, guide procedural decisions, provide insight into anatomy and target localization, and inform patient counseling. This uncertainty is not unique to sensory PNS. In multifidus muscle stimulation for low back pain, which implements a sensorimotor stimulation approach, patient selection often relies on physical examination findings or imaging markers of multifidus muscle dysfunction, yet the predictive value of these diagnostic criteria also remains unclear. Similarly, in occipital neuralgia, occipital nerve blocks have no prognostic value in predicting response to occipital nerve stimulation [[13], [14], [15]] though some found evidence to the contrary [16].

## 6 Central modulation or observational artifact? interpreting study findings within a mechanistic framework

The authors suggest that broader stimulation coverage may drive central modulation through sustained afferent input, potentially explaining the longer-lasting pain relief observed after temporary PNS. While the durability of analgesia after lead removal has been concordant with prior studies [[17], [18], [19]] the mechanism underlying this effect remains uncertain. Central “reconditioning” is an appealing explanation, but in the absence of direct evidence, it remains largely theoretical [20]. Other, more likely explanations such as regression to the mean and placebo effects may also contribute. This is particularly relevant in a cohort selected for moderate-to-severe baseline pain, where some degree of improvement over time is expected regardless of intervention. Disentangling true neuromodulatory effects from these competing explanations will require more rigorous mechanistic studies, ideally incorporating objective biomarkers and sham-controlled blinded designs.

## 7 Conclusion

In summary, this *post-hoc* RESET analysis brings into focus an overlooked question in neuromodulation: how do well-selected patients respond when PNS therapy is delivered as intended for low back pain? The simple answer is that patients who adhere to the treatment protocol stand to benefit the most. However, this conclusion should be tempered by the recognition that adherence is not randomly assigned and likely reflects a constellation of patient-level factors that may independently influence outcomes. Moving forward, the field must look beyond patient selection alone and more closely examine how therapy is delivered, sustained, and optimized over time. As clinicians, researchers, engineers, and policymakers, how should we effectively support and enable patients to remain adherent to therapy in real-world settings? Addressing this will require not only technical refinement, but also more robust study designs and a deeper integration of patient education, expectation-setting, and behavioral support strategies into neuromodulation care pathways.

## Author contributions

RSD, DH, SMH were involved in conception and manuscript composition.

## Funding

The authors have not declared a specific grant for this research from any funding agency in the public, commercial, or not-for-profit sectors.

## Conflicts of interest

RSD received investigator-initiated research grant funding from Nevro Corp and Saol Therapeutics paid to his institution. Other authors declare no conflict of interest.
